# Synthesis and Characterization of Nano-Hydroxyapatite Obtained from Eggshell via the Hydrothermal Process and the Precipitation Method

**DOI:** 10.3390/molecules28134926

**Published:** 2023-06-22

**Authors:** Shih-Ching Wu, Hsueh-Chuan Hsu, Hsueh-Fang Wang, Shu-Ping Liou, Wen-Fu Ho

**Affiliations:** 1 Department of Dental Technology and Materials Science, Central Taiwan University of Science and Technology, Taichung 40601, Taiwan; scwu@ctust.edu.tw (S.-C.W.); hchsu@ctust.edu.tw (H.-C.H.); 2 Department of Nutrition, Hungkuang University, Taichung 43302, Taiwan; fang54@hk.edu.tw; 3 Department of Materials Science and Engineering, Da-Yeh University, Changhua 515006, Taiwan; 4 Department of Chemical and Materials Engineering, National University of Kaohsiung, Kaohsiung 81148, Taiwan

**Keywords:** hydroxyapatite, hydrothermal process, eggshell, bioactivity

## Abstract

Hydroxyapatite (HA) is a major component of the inorganic minerals in the hard tissues of humans and has been widely used as a biomedical ceramic material in orthopedic and dentistry applications. Because human bone contains several impurities, including carbonates, chlorides, fluorides, magnesium, and strontium, human bone minerals differ from stoichiometric HA. Additionally, natural bone is composed of nano-sized HA, and the nanoscale particles exhibit a high level of biological activity. In this paper, HA is prepared via the hydrothermal process because its reaction conditions are easy to control and it has been shown to be quite feasible for large-scale production. Therefore, the hydrothermal process is an effective and convenient method for the preparation of HA. Furthermore, eggshell is adopted as a source of calcium, and mulberry leaf extract is selectively added to synthesize HA. The eggshell accounts for 11% of the total weight of a whole egg, and it consists of calcium carbonate, calcium phosphate, magnesium carbonate, and organic matter. Eggshell contains a variety of trace elements, such as magnesium and strontium, making the composition of the synthesized HA similar to that of the human skeleton. These trace elements exert considerable benefits for bone growth. Moreover, the use of eggshell as a raw material can permit the recycling of biowaste and a reduction in process costs. The purpose of this study is to prepare HA powder via the hydrothermal method and to explore the effects of hydrothermal conditions on the structure and properties of the synthesized HA. The room-temperature precipitation method is used for the control group. Furthermore, the results of an immersion test in simulated body fluid confirm that the as-prepared HA exhibits good apatite-forming bioactivity, which is an essential requirement for artificial materials to bond to living bones in the living body and promote bone regeneration. In particular, it is confirmed that the HA synthesized with the addition of the mulberry leaf extract exhibits good in vitro biocompatibility. The morphology, crystallite size, and composition of the carbonated nano-HA obtained herein are similar to those of natural bones. The carbonated nano-HA appears to be an excellent material for bioresorbable bone substitutes or drug delivery. Therefore, the nano-HA powder prepared in this study has great potential in biomedical applications.

## 1. Introduction

Various bone graft materials are applied to bone defects to accelerate the bone healing process, including the autologous bone graft, allogeneic bone graft, animal bone graft, and artificial synthetic bone graft substitute. Among them, chemically synthesized bioceramics, such as hydroxyapatite (HA, Ca_10_(PO_4_)_6_(OH)_2_) and tricalcium phosphate (TCP, Ca_3_(PO_4_)_2_), have become important bone graft materials [1]. Many studies have explored the synthesis method and properties of HA, but it has the problem of being an expensive, complicated, and time-consuming process. In recent years, the synthesis of HA from natural resources has become a potential route to obtaining high-performance HA powders. However, there are still few studies focusing on the simple and cost-effective processing of HA via using biowaste materials. Therefore, it is worth developing technologies for the preparation of HA using biowaste.

HA is the main mineral component of hard tissues such as bones and teeth, and it exhibits excellent bioactivity, biocompatibility, and osteoconductivity [2]. HA is non-toxic and does not produce an inflammatory reaction in the body, so it is widely used in bone-filling materials, tissue engineering scaffolds, and drug carriers [3]. The ideal stoichiometric Ca/P molar ratio of pure crystalline HA is 1.67. Non-stoichiometric HA may be deficient in calcium or phosphorus. For example, carbonate-substituted apatite structures exhibit higher or lower Ca/P ratios, while HA containing trace elements (Na, Mg, Sr, Fe, Al, Zn, etc.) usually has a lower Ca/P ratio. An appropriate amount of carbonate, a suitable nanoscale morphology, and a non-stoichiometric Ca/P ratio of HA all contribute to its good biocompatibility and biodegradability [4]. The presence of trace elements in HA has also been proven to enhance and accelerate bone growth [5]. Human bone is a nano-grade HA with low degree of crystallinity, and it contains carbonates and a variety of trace elements [3]. Therefore, many researchers have attempted to develop bone-like HA to enhance biocompatibility [6,7].

Wu et al. [6] prepared bone-like HA via the precipitation method from eggshell, followed by a pore former and a sintering procedure to obtain porous biphasic calcium phosphate granules. Experimental results showed that the granules had high biocompatibility and no cytotoxicity. In addition, results from an animal experiment demonstrated that this product had excellent osteoinductivity. Xu et al. [8] established a physiological environment similar to bone matrix vesicles using Dulbecco’s Modified Eagle Medium for biomimetic mineralization (bone-like HA) on alginate microspheres. The results indicated that bone-like HA anchored on alginate microspheres may be a good prospect for bone repair.

Nano-HA crystals are easily decomposed and absorbed in the body because of their large surface areas and weak inter-crystalline bonds [9]. In addition, the large surface area contributes to better sintering at high temperatures, resulting in better mechanical strength and fracture toughness [10]. Moreover, nanocrystalline HA is similar in structure to bone apatite and has good bioactivity, osteoblast adhesion, osteoblast proliferation, and osseointegration. Mei et al. [11] constructed a micro/nano-HA coating on a titanium surface which showed greater bioactivity and biocompatibility under an in vitro culture of MC3T3-E1 osteoblasts. Pilloni et al. [12] explored the behavior of human alveolar osteoblasts on a nano-HA substrate and indicated that due to the increased levels of expression of bone morphogenetic proteins and osteoinductive biomarkers, nano-HA may stimulate the proliferation and differentiation of osteoblasts, thereby promoting bone regeneration at the site of alveolar bone regeneration. Thus, nano-HA particles have become an important research topic in recent years [11,12,13,14,15].

Natural biowaste materials such as oyster shells [16,17], abalone shells [18], and shellfish [19] have been used to synthesize HA. The use of biowaste materials can not only reduce costs but also produce HA containing trace elements and minerals. The practice of converting biowaste materials into HA holds promise for not only addressing the environmental problems caused by waste disposal but also for creating economic benefits. In particular, the trace elements and minerals from biowaste can promote HA to display better bone regeneration performance. There are many methods for preparing HA, such as the precipitation process [20], hydrothermal reaction [21], and microwave method [22]. The principle of the precipitation process is to add a solution containing PO_4_^3−^ to a solution prepared from biowaste material containing Ca^2+^ until the nanoparticles are formed in the form of precipitates. The advantage of this method is the possibility of obtaining a pure and homogeneous product. The principle of the hydrothermal reaction is to prompt the precursor solution to react under high temperature and pressure in a closed reaction environment. The advantage of the hydrothermal method is that the prepared nanoparticles have a uniform morphology and good dispersion. The principle of the microwave method is that polar molecules and ions in the reaction medium selectively absorb microwave radiation energy, thereby generating heat to accelerate chemical reactions. Microwave synthesis has the advantages of rapid and uniform heating and very efficient energy conversion. Among these methods, both the precipitation process and the hydrothermal reaction are commonly used to prepare HA as these techniques are simple processes and can be used to synthesize HA particles with a uniform morphology and size.

Eggshell was used as a source of calcium for the preparation of HA in this study. The main component of eggshell is calcium carbonate (94%), and it also contains calcium phosphate (1%), magnesium carbonate (1%), and organic matter (4%) [23]. Thus, eggshell can also provide other important trace elements. Eggshell was explicitly chosen as the calcium source in this study because of its extremely high calcium content and easy availability, as well as the potential benefits of its trace elements. In this study, HA powder was prepared via the hydrothermal method. The effects of hydrothermal conditions on the structure and properties of the synthesized HA were investigated, and the room-temperature precipitation method was used for the control group. Additionally, mulberry leaf extract was selectively added as the template during the preparation of HA to explore its effect on the properties of the as-synthesized HA.

## 2. Results and Discussions

### 2.1. Microstructure and Morphology of the As-Prepared HA Powder

**Figure 1:**
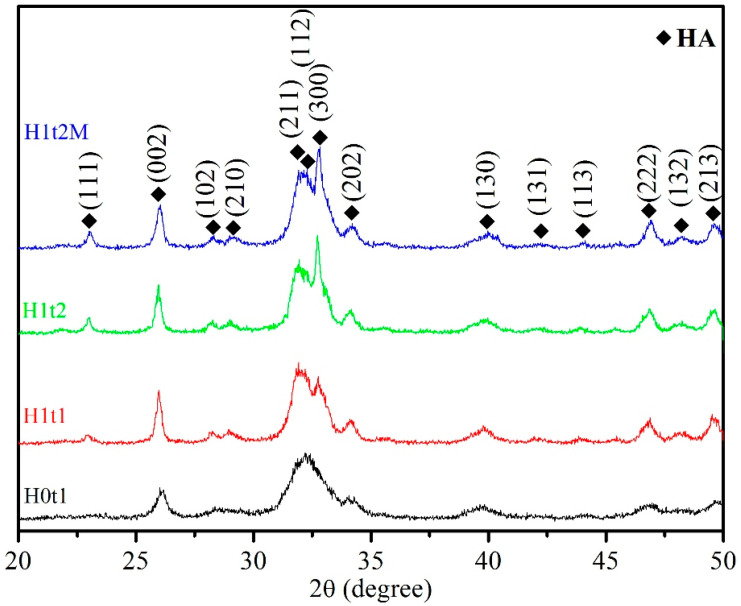
XRD patterns of HA synthesized using the precipitation method at room temperature for 1 h (H0t1), the HA synthesized via the hydrothermal method at 150 °C for 1 h (H1t1) and 24 h (H1t2), and the HA synthesized with the addition of the mulberry leaf extract via the hydrothermal method at 150 °C for 24 h (H1t2M).

Figure 1 presents the XRD patterns of the HA synthesized via the hydrothermal method (H1t1, H1t2, and H1t2M) and precipitation method (H0t1). According to JCPDS data on HA (No. 09-0432), single-phase HA was detected in the samples synthesized under all conditions, and no other phases and residual raw materials were observed.

Moreover, it was found that the diffraction peaks of the HA powder produced via the precipitation method (H0t1) were broader than those of the HA prepared via the hydrothermal method (H1t1, H1t2, and H1t2M), indicating that the powder prepared via the hydrothermal reaction exhibited a higher degree of crystallinity. The degree of crystallinity of the HA powder synthesized via the precipitation method was generally poor [24].

The levels of crystallinity of the synthesized HA particles were calculated from the XRD patterns. The crystallinities of H0t1, H1t1, H1t2, and H1t2M were 25.8%, 44.2%, 51.0%, and 46.1%, respectively. The results showed that the HA (H1t1, H1t2, and H1t2M) fabricated via the hydrothermal method had a greater degree of crystallinity than the HA (H0t1) synthesized via the precipitation method. In the prepared solution, calcium, phosphorus, and hydroxide ions reacted to form HA particles of various sizes. Under the room-temperature precipitation method, the HA particles exhibited less crystallinity and stability. The relatively high-temperature and high-pressure hydrothermal reaction can promote the dissolution, nucleation, and recrystallization of these particles to form a phase with a higher level of crystallinity. Moreover, with the increase in the hydrothermal reaction time, the crystallinity of the HA increased [25,26]. In addition, the level of crystallinity of the HA (H1t2M) synthesized by adding the mulberry leaf extract was lower than that of H1t2 under the same hydrothermal conditions, which was probably due to crystal imperfections [27]. The dissolution rate of a powder is strongly correlated with its level of crystallinity. The low crystallinity of HA has a faster dissolution ability, which can facilitate its bonding with adjacent bones [28]. Factors affecting the crystallinity of the nano-HA synthesized in this study include the synthesis methods, the parameters of hydrothermal reaction, and the addition of the mulberry leaf extract.

The crystallite sizes of the HA particles were calculated using the Scherrer equation. The crystallite sizes of H0t1, H1t1, H1t2, and H1t2M were 21.0, 26.5, 40.8, and 25.5 nm, respectively. The crystallite sizes of the HA synthesized via the hydrothermal reaction were larger than those synthesized via the precipitation method. Additionally, as the hydrothermal reaction time increased, the crystallite size also increased significantly. Furthermore, with the addition of the mulberry leaf extract, HA with a slightly smaller crystallite size was obtained. The HA synthesized in this experiment was similar to human bone apatite, which is a nano-crystalline HA [29].

**Figure 2:**
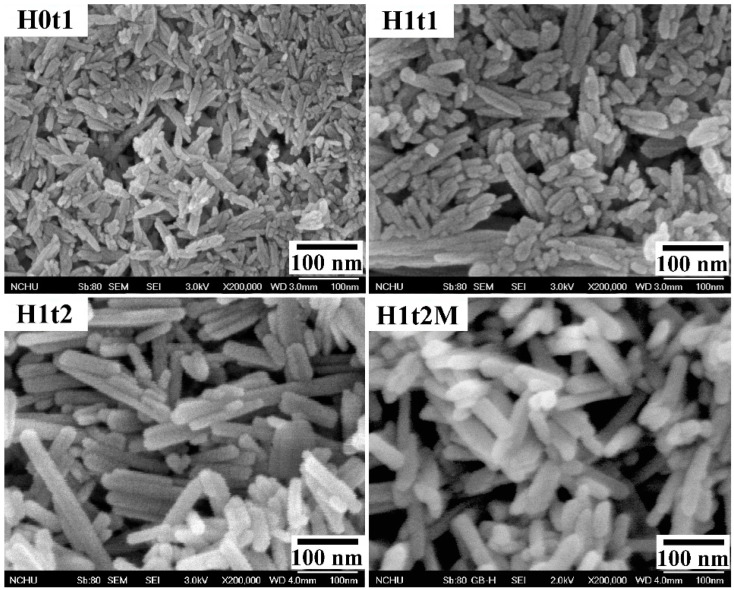
FE-SEM images of HA synthesized using the precipitation method at room temperature for 1 h (H0t1), using the hydrothermal method at 150 °C for 1 h (H1t1) and 24 h (H1t2), and with the addition of the mulberry leaf extract, using the hydrothermal method at 150 °C for 24 h (H1t2M).

Figure 2 shows FE-SEM images of the HA samples synthesized using the precipitation method at room temperature for 1 h (H0t1), using the hydrothermal method at 150 °C for 1 h (H1t1) and 24 h (H1t2), and with the addition of the mulberry leaf extract, using the hydrothermal method at 150 °C for 24 h (H1t2M). The HA samples synthesized using the precipitation and hydrothermal methods comprised rod-shaped nanoparticles. According to the FE-SEM images, the average particle lengths of H0t1, H1t1, H1t2, and H1t2M were calculated to be 78, 126, 198, and 111 nm, respectively. The results reveal that the particle lengths of the HA prepared using the hydrothermal method were significantly longer than those synthesized using the precipitation method. Moreover, with the increase in the hydrothermal reaction time, the particle lengths of the HA became far longer. During the reaction, the HA particles grew along the preferred c-axis direction, so the lengths of the HA particles increased with the increase in the hydrothermal reaction time [30]. In addition, when the mulberry leaf extract was added, the lengths of the HA particles became shorter because the additive hindered the binding of calcium, phosphorus, or hydroxide ions during the reaction and affected the growth of the HA grains [31].

### 2.2. Characterization of the As-Prepared HA Powder

**Figure 3:**
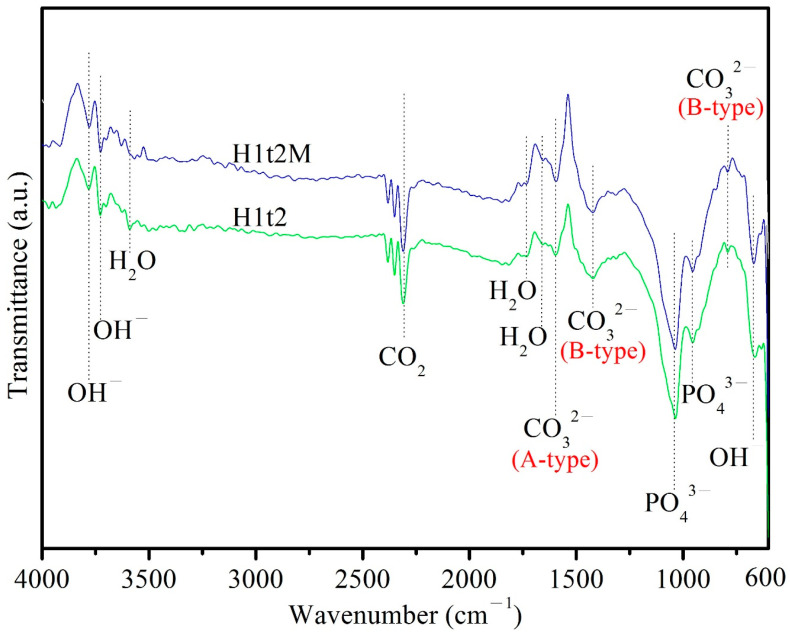
ATR-FTIR spectra of HA synthesized using the hydrothermal method at 150 °C for 24 h, with (H1t2M) and without (H1t2) the addition of the mulberry leaf extract.

Figure 3 depicts the ATR-FTIR spectra of the HA samples synthesized using the hydrothermal method at 150 °C for 24 h with (H1t2M) or without (H1t2) the addition of the mulberry leaf extract. The peak at 671 cm^−1^ corresponded to the bending vibrations of OH^−^, and the peaks at 3720 and 3784 cm^−1^ corresponded to the stretching vibrations of OH^−^. The bands at 958 and 1040 cm^−1^ corresponded to the stretching vibrations of the PO_4_^3−^ groups. The peaks at 1665, 1733, and 3590 cm^−1^ corresponded to H_2_O adsorbed on the as-synthesized HA [32]. The bands at 790 and 1422 cm^−1^ corresponded to the bending and stretching vibrations of CO_3_^2−^, which replaced PO_4_^3−^ in the HA; this is referred to as a B-type carbonate-containing HA [31]. The peak at 1596 cm^−1^ was attributed to the stretching vibrations of CO_3_^2−^, which replaced the OH^−^ site in the HA; this is referred to as A-type carbonate-containing HA [33]. Therefore, the HA synthesized in this experiment is an AB-type carbonate-substituted HA, which is commonly found in natural bone [34]. In addition, the Ca/P ratio of the AB-type carbonate-substituted HA would deviate from 1.67, so it exhibits a higher level of bioactivity and better sintering quality than stoichiometric HA [35].

The compositions of the HA synthesized using the hydrothermal method at 150 °C for 24 h with (H1t2M) and without (H1t2) the addition of the mulberry leaf extract were analyzed using ICP-AES (Table 1). The results indicated that Mg and Sr were the main trace elements in the HA, which were mainly derived from the eggshell [36]. Mg is beneficial for bone metabolism and can stimulate new bone formation. Furthermore, it is responsible for the adhesion and stability of osteoblasts [37]. Meanwhile, Sr indicates a generalized improvement in bone regeneration and can reduce the risk of bone fracture [38]. In the past few years, several researchers have incorporated Sr or Mg into bioceramics, such as calcium phosphates and bioactive glasses, to enhance bone formation [24,39].

**Figure 4:**
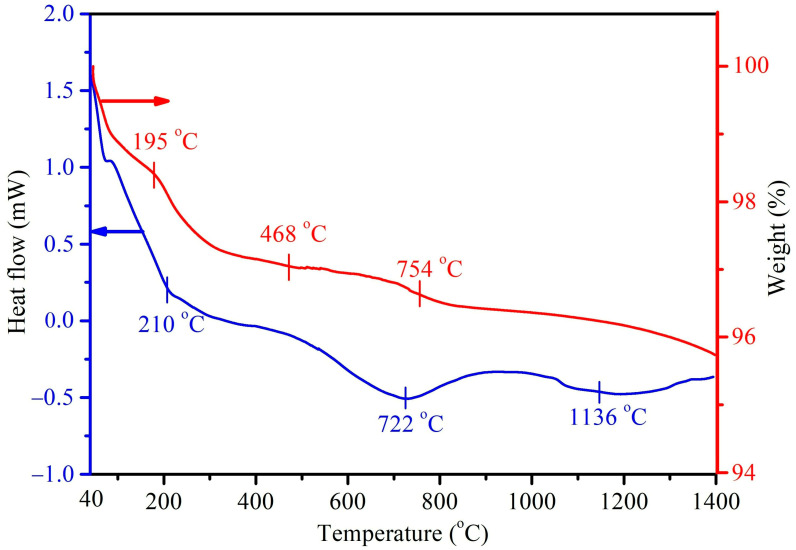
DTA/TGA thermograms of the HA prepared using the hydrothermal method at 150 °C for 24 h without the addition of the mulberry leaf extract (H1t2).

Figure 4 shows the DTA/TGA thermograms of the as-prepared HA without the mulberry leaf extract (H1t2), prepared using the hydrothermal method at 150 °C for 24 h. In this study, DTA measurements were mainly conducted to investigate the endothermic/exothermic reactions or phase changes of the prepared HA samples. Three major endothermic peaks at 210, 722, and 1136 °C were observed in the DTA curve. The first endothermic peak at 210 °C corresponded to the desorption of physically adsorbed water and with further heating up to 500 °C, possibly brought about the removal of interstitial water molecules in the crystal lattice. The endothermic peak at 722 °C could be assigned to the decarboxylation of the HA sample, as well as the phase transformation of HA into β-TCP. The last endothermic peak at 1136 °C was mainly attributed to the phase change of β-TCP into α-TCP. Viswanath et al. [40] prepared HA via the hydrothermal method and found that the temperature for the phase change from HA to β-TCP was 743 °C, indicating that the Ca-deficient HA had a lower phase change temperature. On the other hand, the DTA curve of the HA (H1t2M) synthesized via the addition of the mulberry leaf extract was similar to that of its counterpart H1t2 (without the mulberry leaf extract), and it revealed three main endothermic peaks. The TGA results showed that the weight change in H1t2 at 40–195 °C was due to the evaporation of surface-adsorbed water molecules, and the weight change at 195–468 °C was related to the removal of crystallization water. The weight loss at a temperature range of 650–800 °C possibly corresponded to the decarboxylation of the HA.

### 2.3. Bioactivity and Biocompatibility of the As-Prepared HA Sample

**Figure 5:**
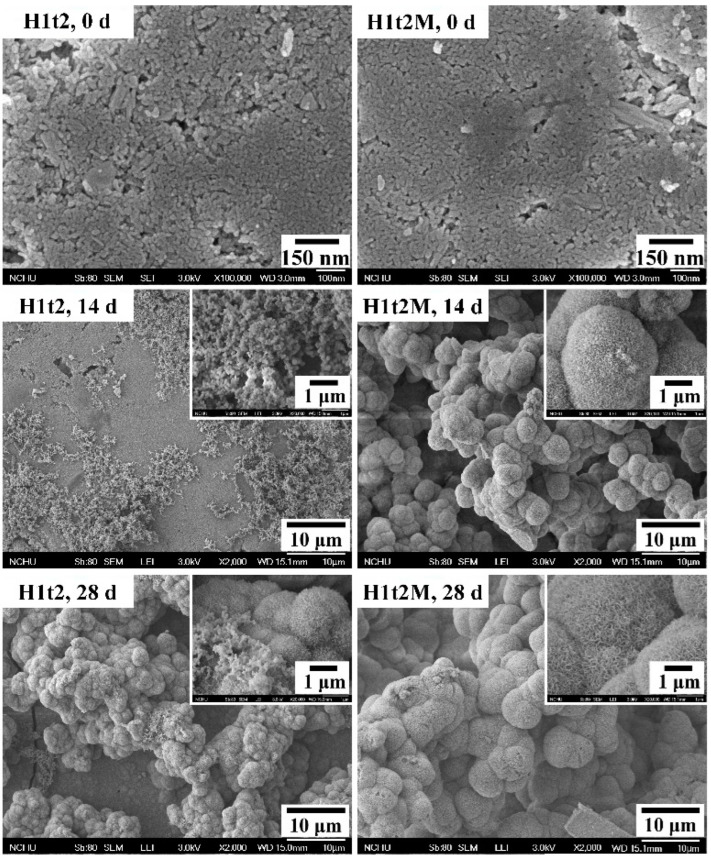
FE-SEM images of HA synthesized using the hydrothermal method at 150 °C for 24 h with (H1t2M) and without (H1t2) the addition of the mulberry leaf extract before and after soaking in simulated body fluid (SBF) for 14 and 28 days.

In order to evaluate the bioactivity of the HA synthesized in this experiment, the disk-shaped sample were immersed in SBF, and the apatite phases formed on the surfaces of the samples were observed. In this assessment, the HA samples with (H1t2M) and without (H1t2) the mulberry leaf extract prepared using the hydrothermal method at 150 °C for 24 h were immersed in SBF for 14 or 28 days, followed by SEM observation, as shown in Figure 5.

After 14 days of soaking in SBF, several granular precipitates were found on the surfaces of H1t2 and H1t2M, and the apatite precipitates on the surface of H1t2M were significantly larger. After soaking in SBF for 28 days, the surfaces of both samples were entirely covered with spheroidal precipitates. It can be seen in the high-magnification images that the apatite precipitates were composed of flaky or plate-like crystals. Therefore, the HA samples prepared in this study via the hydrothermal method with and without the mulberry leaf extract demonstrated better bioactivity, which is an essential requirement for artificial materials to bond to living bones in the living body [41]. Since SBF has similar ion concentrations to the inorganic components in human plasma, biomineralization studies in SBF can demonstrate the similarity between the in vitro and in vivo behavior of bioceramics. The ability to form an apatite layer on the surface of synthetic HA is positively related to the bioactivity of HA.

**Figure 6:**
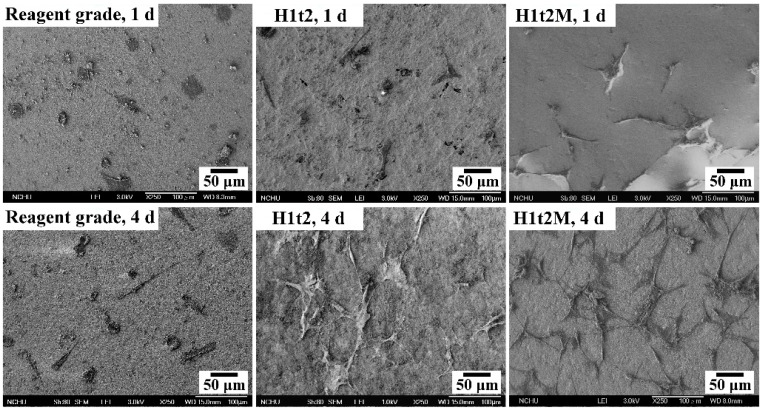
FE-SEM images of the MG63 osteoblast-like cells cultured on the surfaces of disk-shaped HA samples synthesized using the hydrothermal method at 150 °C for 24 h with (H1t2M) and without (H1t2) the addition of the mulberry leaf extract after culturing for 1 and 4 days. Commercially available Ca-deficient HA (reagent grade) was used as the control group.

The samples of HA synthesized via the hydrothermal reaction at 150 °C for 24 h with (H1t2M) and without (H1t2) the addition of the mulberry leaf extract were subjected to cell culturing for 1 and 4 days. A commercially available reagent-grade Ca-deficient HA (Shimakyu’s Pure Chemicals Co., Osaka, Japan) was used as the control group. Figure 6 presents FE-SEM images of MG63 osteoblast-like cells cultured on the surfaces of disk-shaped HA samples after culturing for 1 and 4 days. The results showed that the cells exhibited good adhesion on the surfaces of H1t2, H1t2M, and reagent-grade HA. However, the photographs of the cell culture, taken for 4 days, showed that the cells on the surfaces of the H1t2 and H1t2M samples had obvious filopodia, a positive sign for the better biocompatibility of the HA prepared in this study.

## 3. Materials and Methods

The waste eggshells were washed with distilled water to remove the egg membranes and adhesions. They were then dried overnight at 80 °C and finally ground into powder. An appropriate amount (2.0 g) of eggshell powder was added to a 25 vol% aqueous solution of HCl (20 mL), and the eggshell powder was dissolved in it by stirring for 1 h to complete the dissolution of the shell. Then, 85 wt% H_3_PO_4_ (0.85 mL) was added dropwise to produce a Ca/P molar ratio of 1.67. After adjusting the pH to 10 with ammonia, the solution was uniformly stirred and poured into a Teflon container. The vessel was then placed in a stainless-steel autoclave for the hydrothermal reaction at 150 °C for 1 h or for 24 h (denoted as H1t1 and H1t2, respectively). The heating rate was fixed to 10 °C/min, and the autoclave was cooled in air to room temperature after heating. In this experiment, an aqueous solution of 1 wt% mulberry leaf extract (10 mL) was selectively added to the above reaction solution for the synthesis of HA under hydrothermal conditions at 150 °C for 24 h (H1t2M). Additionally, a reaction solution prepared via the same procedure described above was used to synthesize HA at room temperature for 1 h via the precipitation method, and this procedure was assigned as the control group (H0t1). After the hydrothermal reaction or the precipitation method, an HA suspension was obtained. After the HA was washed five times repeatedly with distilled water, the precipitate was isolated via suction filtration, followed by drying at 80 °C for 24 h and grinding to obtain HA powders.

The crystal phases of the synthesized HA powders were analyzed using X-ray diffraction (XRD; XRD-6000, Shimadzu, Japan) with Cu Kα radiation. The operating voltage was 30 kV, the current was 30 mA, and the scan rate was 2°/min. The crystallinity (Xc) of the as-synthesized HA was calculated using [42]:

(1) $$X_{c}=1-\frac{V_{112/300}}{I_{300}}$$

where V_112/300_ is the intensity of the hollow between the (112) and (300) diffraction peaks and I_300_ is the intensity of the (300) diffraction peak. The crystallite sizes of the HA particles were calculated using the Scherrer equation from the XRD data [43]:

(2) $$X_{s}=\frac{0.9\lambda }{\mathrm{FWHM}\;\cos\theta }$$

where X_s_ is the average crystallite size (nm), λ is the wavelength of X-ray radiation (0.15406 nm), FWHM is the full-width at half-maximum intensity of the (002) diffraction peak (rad), and θ is the Bragg angle (degree).

In this study, field-emission scanning electron microscopy (FE-SEM; JSM-6700F, JEOL, Japan) was used to observe the morphologies of the HA powders. FE-SEM images with 200,000× magnification were recorded to calculate the HA particle sizes. A total of thirty randomly selected individual particles were taken from three photographs of each sample to obtain an average value. The functional groups of the HA samples were analyzed using Fourier-transform infrared spectroscopy (ATR-FTIR; Bio-Rad, FTS-40, Hercules, CA, USA). A thermal analysis of the HA samples was performed under a nitrogen atmosphere using differential thermal analysis/thermogravimetric analysis (DTA/TGA; SDT Q600, TA, New Castle, DE, USA) in a range of temperatures from 40 °C to 1400 °C at a heating rate of 10 °C/min. A quantitative analysis of the compositions of the HA samples was performed using inductively coupled plasma atomic–emission spectrometry (ICP-AES; 725, Agilent, Santa Clara, CA, USA).

In this experiment, the levels of bioactivity of the synthesized HA powders were assessed via their immersion in acellular simulated body fluids (SBFs). The HA powders were pressure-formed into disks with diameters of 13 mm, followed by heat treatment at 400 °C for 2 h. The SBF used in this study was the formulation proposed by Kokubo and Takadama [44]. The disk-shaped sample was placed in a 50 mL glass beaker, followed by the addition of 40 mL of the SBF, and then the beaker was sealed using plastic wrap. Next, the beaker was placed in a water bath at 37 °C for 14 or 28 days. During the soaking process, the SBF was replaced once every two days. After soaking, the samples were rinsed with deionized water and dried in an oven at 45 °C.

In this study, human osteoblastic cells (MG-63) were used for the cell culture (human osteoblast-like MG-63 cells were obtained from Taiwan Bioresource Collection and Research Center (BCRC, Number 60279). The synthesized HA powders were prepared into disk-shaped samples with diameters of 13 mm, which was the same as the samples used for the bioactivity test. The front and back sides were sterilized with UV irradiation for 30 min to prepare for cell culturing. The samples were placed in a 24-well culture plate, and the MG-63 cell suspension (1 × 10^4^ cells/mL) was added to the wells, followed by the addition of 2 mL of pure Dulbecco’s Modified Eagle Medium (DMEM) to each well. Then, the culture plate was stored in a 37 °C incubator (5% CO_2_; 95% air) for 1 or 4 days. The cell culture medium was changed every 2 days. After cell culturing, the samples were washed with PBS and then immersed in glutaraldehyde for 24 h to fix the cells, followed by dehydration in a graded ethanol solution and critical point drying. Cell morphology and cell adhesion were observed using FE-SEM.

## 4. Conclusions

In this study, hydroxyapatite (HA) particles were synthesized using eggshell as a source of Ca via the precipitation method (H0t1) and the hydrothermal method (H1t1 and H1t2), and mulberry leaf extract (H1t2M) was selectively added to the reaction solution. The products prepared in this study comprised single-phase HA. The results show that the hydrothermal reaction enhanced the crystallinity of HA, while the addition of the mulberry leaf extract slightly decreased the crystallinity. Nano-sized crystals were observed in the samples of HA synthesized under all conditions. The crystallite size of the HA produced using the hydrothermal method was greater than that produced using the precipitation method, and the size increased with increasing hydrothermal time. The HA fabricated with the addition of the mulberry leaf extract exhibited a smaller crystallite size. Additionally, under all synthesis conditions, rod-shaped HA particles were obtained, and the average length of the HA particles synthesized using the hydrothermal method was longer than that of the HA particles obtained using the precipitation method, and the length increased with the increase in the hydrothermal reaction time. The addition of the mulberry leaf extract could suppress the growth of the c-axis of the HA crystals, and the as-prepared HA thus exhibited a shorter crystal length and wider width. The Fourier-transform infrared spectroscopy results showed that the synthesized HA contained OH^−^, PO_4_^3−^, and CO_3_^2−^ functional groups. Furthermore, it was confirmed that the product was AB-type carbonate-containing HA. The inductively coupled plasma atomic–emission spectrometry analysis indicated that the trace elements in the as-prepared HA were mainly Mg and Sr. The differential thermal analysis/thermogravimetric analysis revealed that HA (H1t2) was changed into β-TCP at 722 °C, and then the β-TCP was transformed into α-TCP at 1136 °C. After soaking the samples in simulated body fluid, the surfaces of the samples (H1t2 and H1t2M) each revealed a layer of low-crystallinity apatite, which was composed of several flakes and plate crystals, and this apatite layer increased when increasing the soaking time. Additionally, the 4-day cell culture showed that human-osteoblast-like MG63 cells on the surfaces of the samples (H1t2 and H1t2M) had obvious filopodia. Therefore, both H1t2 and H1t2M exhibited good bioactivity and biocompatibility. Accordingly, it was confirmed that nano-HA can be synthesized via the hydrothermal method or the precipitation method using eggshell as a source of Ca. Additionally, the bone-like HA, which was an AB-type carbonated structure and contained trace elements, is promising for use in bone repair applications.

## Figures and Tables

**Table 1:** The compositions of the HA synthesized using the hydrothermal method at 150 °C for 24 h with (H1t2M) and without (H1t2) the addition of the mulberry leaf extract were analyzed using ICP-AES.

Title 1	Ca (wt%)	P (wt%)	Mg (wt%)	Sr (wt%)
H1t2	24.700	10.500	0.173	0.019
H1t2M	32.700	16.000	0.321	0.043

## Data Availability

Not applicable.

## Sample Availability

Samples of the compounds are not available from the authors.
